# Insulin Resistance Predicts Severity of Coronary Atherosclerotic Disease in Non-Diabetic Patients

**DOI:** 10.3390/jcm9072144

**Published:** 2020-07-07

**Authors:** Teresa Strisciuglio, Raffaele Izzo, Emanuele Barbato, Giuseppe Di Gioia, Iginio Colaiori, Antonella Fiordelisi, Carmine Morisco, Jozef Bartunek, Danilo Franco, Giuseppe Ammirati, Valerio Pergola, Livio Imparato, Bruno Trimarco, Giovanni Esposito, Antonio Rapacciuolo

**Affiliations:** 1 Department of Advanced Biomedical Sciences, University of Naples Federico II, 80131 Naples, Italy; teresa.strisciuglio@unina.it (T.S.); raffaele.izzo@unina.it (R.I.); giuseppe.digioia88@gmail.com (G.D.G.); antofior@gmail.fr (A.F.); carmine.morisco@unina.it (C.M.); francodnl88@gmail.com (D.F.); giuseppe.ammirati92@gmail.com (G.A.); valerio.pergola.pz@gmail.com (V.P.); livioimparato91@gmail.com (L.I.); trimarco@unina.it (B.T.); giovanni.esposito2@unina.it (G.E.); antonio.rapacciuolo@unina.it (A.R.); 2 Cardiovascular Research Center OLV Hospital, 9300 Aalst, Belgium; i.colaiori@unicampus.it (I.C.); jozef.bartunek@olvz-aalst.be (J.B.)

**Keywords:** coronary atherosclerosis, insulin resistance, cardiovascular prevention

## Abstract

Background: Insulin resistance (IR) in patients with type 2 diabetes mellitus (T2DM) represents a predictor of coronary artery disease (CAD). However, how IR is able to impact the severity of coronary atherosclerosis in non-diabetic patients is unknown. Objectives. We investigated the relation between the IR and the extent and severity of coronary atherosclerosis in non-diabetic patients referred to coronary angiography (CA) Methods: Consecutive patients undergoing to CA for acute coronary syndromes or stable angina were analyzed. The IR was assessed by mean of the homeostasis model assessment of insulin resistance (HOMA-IR) whereas the SYNTAX score (SS) was used as index of the severity of coronary atherosclerosis Results: Overall, 126 patients were included, with a median SS of 12 (IQR 5.25–20.5). Patients were divided in four groups according to the distribution in quartiles of SS (SS1-2-3-4). A significant correlation between HOMA-IR and SS was observed, especially in women. A progressive increase of HOMA-IR was observed in parallel with the increasing severity (from SS1 to SS4) and extension (1-2-3-vessel disease) of coronary atherosclerosis. Multivariable analysis showed that the HOMA-IR was the strongest independent predictor of severe (SS4) and extensive (three-vessel disease) coronary atherosclerosis. Conclusion: Insulin resistance goes hand in hand with the extension and severity of coronary atherosclerosis in non-diabetic patients. The HOMA index is an independent predictor of three-vessel disease at CA. The HOMA index could be useful for risk stratification of CAD even in absence of T2DM.

## 1. Introduction

The major cause of death in patients affected by type 2 diabetes mellitus (T2DM) is cardiovascular disease (CVD) with coronary artery disease (CAD) and stroke being the main contributors [1]. Insulin resistance (IR) is a hallmark of T2DM, however it may precede by many years the clinical diagnosis of T2DM and exert detrimental effects [2,3]. Two decades ago, Pyörälä et al. investigated the relevance of IR syndrome in healthy subjects [4]. The latter, along with other clustering risk factors (i.e., body mass index, mean blood pressure, tryglicerides, subscapular skinfold, levels of insulin and glucose during oral glucose tolerance test), was evaluated in a large population of 970 middle-aged healthy men (34–64 years) followed-up for 22 years and was demonstrated to increase the risk of CAD and stroke [4].

The homeostasis model assessment of insulin resistance (HOMA-IR) [5] is an index based on a mathematical model that considers serum levels of fasting glucose and insulin and because it has been demonstrated to have a high correlation with the hyperinsulinemic-euglycemic clamp it is considered a reliable indicator of IR [6].

Insulin resistance measured by HOMA index was demonstrated to be a risk factor for carotid atherosclerosis in subjects with normal fasting glucose and normal glucose tolerance [7]. Furthermore, Mossmann et al. demonstrated a relation between IR and coronary atherosclerosis in non-obese non-T2DM patients; high HOMA-IR was associated with significant CAD (coronary artery stenoses > 50%) [8]. However, to what extent and how IR influences coronary atherosclerosis in non-diabetic patients with normal fasting glucose and normal glycated hemoglobin (Hb1Ac) is not well established.

In the current study, we sought to investigate the relation between the extent and severity of coronary atherosclerosis and the HOMA-IR in nondiabetic-patients referred to coronary angiography (CA) for acute coronary syndromes (ACS) or stable angina.

## 2. Methods

### 2.1. Study Population

We prospectively analyzed consecutive patients undergoing coronary angiography (CA) for ACS or stable angina in the Catheterization Laboratories of the University of Naples Federico II (Italy) and of the OLV Heart Centrum in Aalst (Belgium) from February 2018 to June 2019. Exclusion criteria were age < 18 years, pregnancy or breastfeeding, and presence of pre-diabetes or overt diabetes. Pre-diabetes and diabetes were diagnosed based on the fasting plasma glucose or the glycated hemoglobin according to the current guidelines (FPG ≥ 100–125 mg/dL and Hb1Ac ≥ 5.6–6.4% for pre-diabetes; FPG ≥ 126 mg/dL and Hb1Ac ≥ 6.5% [9]. Patients’ and procedural characteristics were retrieved from the local databases. All patients signed the informed consent for the procedure and for data collection. This study is in accordance with the Declaration of Helsinki and local ethical committees approved it.

### 2.2. Definitions

Pre-diabetes and diabetes were diagnosed based on the fasting plasma glucose or the glycated hemoglobin according to the current guidelines (Fasting plasma glucose (FPG) ≥ 100–125 mg/dL and glycated hemoglobin (Hb1Ac) ≥ 5.6–6.4% for pre-diabetes; FPG ≥ 126 mg/dL and Hb1Ac ≥ 6.5% [9]. The systolic and diastolic blood pressure (BP) was measured by standard aneroid sphygmomanometer after 5 min rest in the supine position. Three BP measurements were obtained in the sitting position, at 2 min intervals. The averages of these measurements were used for the analysis. Hypertension is defined as office systolic blood pressure (SBP) values of 140 mmHg and/or diastolic BP (DBP) values of 90 mmHg, according to the European Society of Cardiology (ESC) guidelines [10]. The body mass index (BMI) was defined as the body mass divided by the square of the body height, expressed in units of kg/m^2^.

Hypercholesterolemia was defined as serum cholesterol level > 200 mg/dL or the use of lipid-lowering agents [11]. Hypertriglyceridemia was defined as fasting or non-fasting triglycerides (TG) 175–499 mg/dL (1.9–5.6 mmol/L). Dyslipidemia refers to the presence of Hypercholesterolemia and/or hypertriglyceridemia [11]. The self-reported history of diabetes, hypercholesterolemia, hypertension, as well as the self-reported medication use was cross-checked with the records of the general practitioners. The left ventricle ejection fraction (LVEF%), a measure of left ventricular systolic function, was assessed during a comprehensive trans-thoracic echocardiography with the Simpson’s method (biplane method of disks) by tracing the endocardial border in both the apical four-chamber and two-chamber views in end-systole and end-diastole.

The renal function was assessed by the CKD-EPI (Chronic Kidney Disease Epidemiology Collaboration) equation easily accessible through a web-calculator.

### 2.3. Evaluation of the Complexity and the Extent of the Coronary Atherosclerosis

SYNTAX score I (SS) was used as an index of the severity of coronary atherosclerosis [12,13] and was measured by reviewing the cine-loop recordings of every procedure by two independent interventional cardiologists, that were blinded to the HOMA-IR of the patients. This score takes into account 11 variables: Dominance, number of diseased segments, total occlusion, trifurcation, bifurcation type and angulation, aorto-ostial lesion, tortuosity, lesion length, heavy calcification, thrombus, diffuse disease [12].

When the SS differed between the cardiologists, they had to reach a consensus by reviewing together the cine-loop recordings. The extent of coronary atherosclerosis was measured based on the number of coronary vessels with significant atherosclerotic lesions ≥ 50%. Three-vessel disease means that the three main vessels (right coronary artery, left anterior descending artery, and the circumflex artery) had significant coronary lesions.

### 2.4. Assessment of Insulin Resistance

Blood samples were obtained in stable hemodynamic conditions the day before the discharge from the hospital in order to measure fasting glucose and insulin levels and therefore to calculate the HOMA-IR with the following formula HOMA index = [fasting insulin × fasting glucose] / 22.5. Results of the HOMA-IR were divided into percentiles and a value ≥ the 75th percentile was considered pathologic [14,15].

### 2.5. Statistical Analysis

The data were expressed as mean ± 1 standard deviation (SD) and the variables that were not normally distributed were log-transformed. Normality of distribution was tested by means of the nonparametric Kolmogorov–Smirnov test. The ANOVA analysis was used to compare the baseline continuous variables of the four groups of patients according to SS. Under the assumption of increasing abnormalities from group 1 to group 4, polynomial linear and quadratic contrasts were used to estimate the trend. The Χ^2^ distribution was used to compare the categorical variables, with the Monte Carlo simulation in order to obtain exact p-values. The Pearson correlation was performed to assess the correlation between the SS and the HOMA-IR. Partial correlation was used to assess the influence of other variables on the correlation between SS and HOMA-IR. Binary logistic regression analysis was performed to identify predictors of severe (SS4) and extensive (3-vessel) coronary atherosclerosis. Adjustment for confounding variables was obtained by including simultaneously all the variables in the regression analysis. The variable three-vessel disease has been used as a dichotomous variable in the binary logistic regression. Non-parametric regression with natural cubic splines was used to depict the correlation between HOMA-IR and SS (continuous variable). Sensitivity, specificity, and optimal diagnostic cut-off value for the HOMA-IR were defined from the calculated receiver operator characteristic (ROC) curves, as appropriate. The optimal diagnostic cut-off value was defined based on the Youden’s index, calculated as [(sensitivity + specificity) – 1], namely, where the sum of sensitivity and specificity is maximized. All statistical analyses were performed by means of SPSS 25.0 (IBM, Armonk, NY, USA).

## 3. Results

### 3.1. Patients’Characteristics

One hundred and twenty-six patients out of 200 undergoing CA were included in the analysis, whereas 74 were excluded because of pre-diabetes or diabetes. The SS was not normally distributed; thus, the values were log-transformed. The median SS was 12 (IQR 5.25–20.5) and the mean ± SD was 14.3 ± 10.7. Patients were divided in four groups (SS1-2-3-4) according to the distribution in quartiles of SS, with the highest quartile (SS4) indicating patients with high SS, therefore severe coronary atherosclerosis. In Table 1, the baseline clinical characteristics are reported. No significant differences were found between groups.

### 3.2. Insulin Resistance and its Relation with the Severity and the Extension of Coronary Atherosclerosis

**Figure 1:**
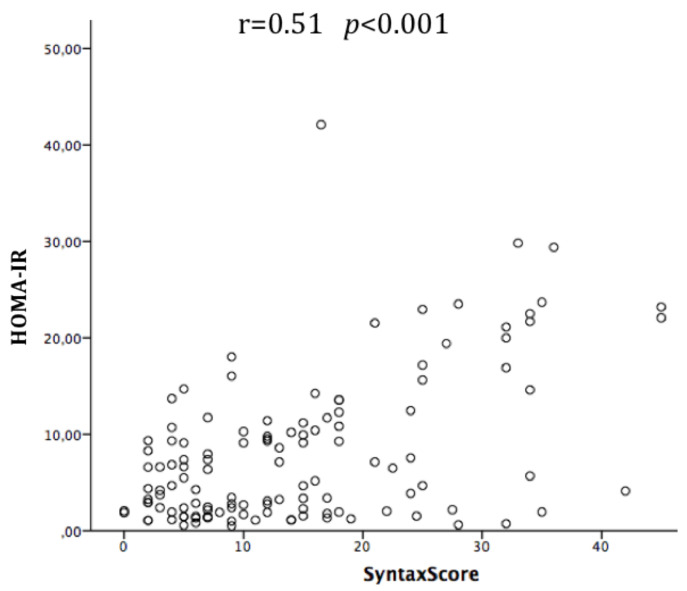
Correlation between syntax score and homeostasis model assessment of insulin resistance (HOMA-IR) in the overall population.

**Figure 2:**
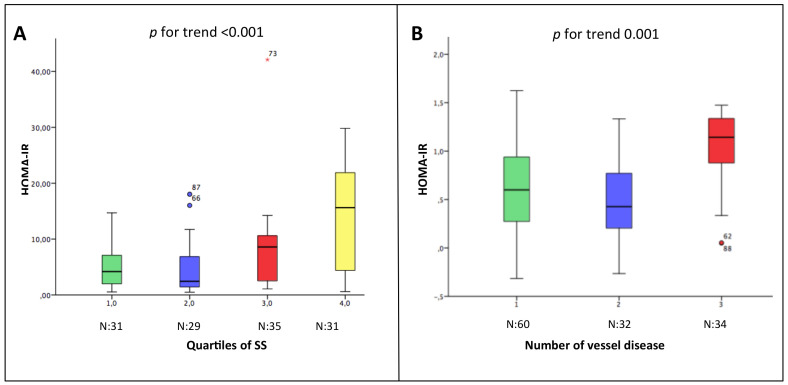
Relation between the HOMA-IR and the severity (Panel **A**) and the extension (Panel **B**) of coronary atherosclerosis. In Panel **A**, patients are divided per tertiles of Syntax Score. In Panel **B**, patients are divided based on the number of vessel disease. * Outliers.

**Figure 3:**
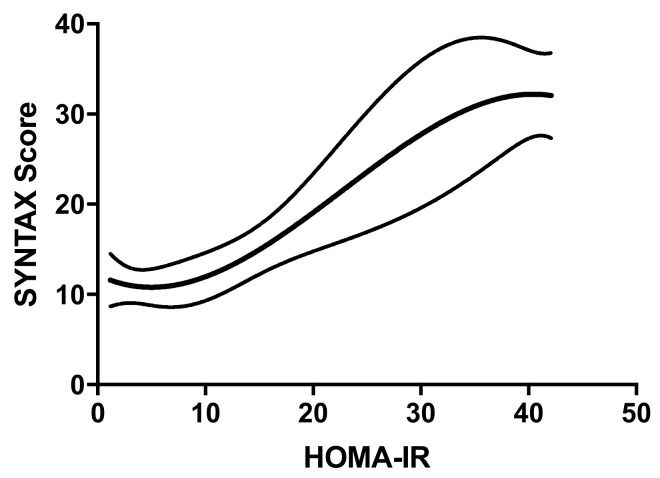
Plot of the non-parametric regression with natural cubic splines, showing the correlation between HOMA-IR and SS. This plot shows the correlation between HOMA-IR and SS. The upper and lower lines represent the 95% confidence intervals (CI). The equation used is the following: SS = 12.19 − 0.5812 * HOMA-IR + 0.06551 * (HOMA-IR)2 −0.0009619 * (HOMA-IR)3.

The HOMA-IR was not normally distributed; thus, the values were log-transformed. The median HOMA-IR was 5.15 (IQR 1.98–10.99) and the mean ± SD was 7.9 ± 7.6. A significant and moderate correlation between HOMA-IR and SS was observed in the overall population (r = 0.51, *p* < 0.001) (Figure 1). The correlation was stronger for women than men (r = 0.77 *p* < 0.001 vs. r = 0.44 *p* < 0.001). The partial correlation analysis showed that none of the baseline variables (gender, age, BMI, diabetes, hypertension, dyslipidemia, use of cardiovascular drugs (Ca-antagonists/Beta-blockers/ACE-ARBs/Statins) influenced the correlation between HOMA-IR and SS. A progressive increase of HOMA-IR was observed in parallel with the increasing severity (from SS1 to SS4) and extension (1-2-3-vessel disease) of coronary atherosclerosis (Figure 2). The same results were observed in both genders. Even when considering the SS as a continuous variable (Figure 3), we observed that, as the HOMA-IR increased, there was a concordant increase in the SS.

Furthermore, patients with HOMA-IR ≥ the 75th percentile had a significantly higher mean SS than those < 75th percentile (*t*-test: 23.6 ± 11.1 vs. 11.2 ± 8.6. *p* < 0.001).

### 3.3. Predictors of Severity and the Extension of Coronary Atherosclerosis

**Figure 4:**
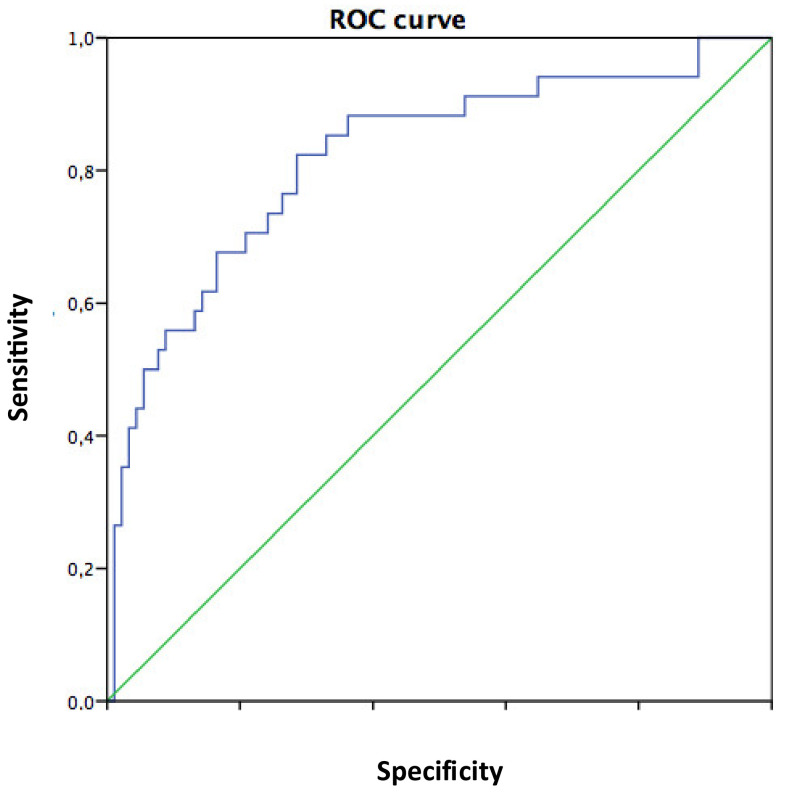
ROC curve analysis. Ability of the HOMA-IR to predict three-vessel disease. The AUC is 0.82. The area under the curve (AUC) is 0.82. The HOMA-IR has a good accuracy in predicting patients with or without three-vessel disease.

Binary logistic regression analysis showed that the HOMA-IR was the strongest independent predictor of severe (SS4) and extensive (three-vessel disease) coronary atherosclerosis (Table 2: panel A and B, respectively). Furthermore, the ROC curve analysis showed that a HOMA-IR value of 10.22 or above had a high sensitivity (62%) and specificity (85%) to identify non-diabetic patients at risk of having three-vessel disease (AUC 0.82) (Figure 4).

## 4. Discussion

### 4.1. Main Findings

The current study showed, for the first time, that in non-diabetic patients: (1) There is a moderate correlation between HOMA-IR and SS, especially in women; (2) insulin resistance goes hand in hand with the extent and the severity of coronary atherosclerosis; (3) HOMA-IR is a strong and independent predictor of severe CAD and of three-vessel disease; and (4) A HOMA-IR value of 10.22 or above should be considered a warning mark in non-diabetic patients as they may have a severe atherosclerosis involving the three coronary arteries. 

### 4.2. Insulin Resistance as a Risk Factor for CVD in Non-Diabetic Subjects

Cardiovascular disease accounts for more than a half of all deaths occurred between 2007 and 2017 among T2DM patients all over the world [1], with CAD and stroke being the most common causes.

Insulin resistance is a hallmark of T2DM and contributes to the increased CV risk of diabetic patients [15], however its association with CVD is also independent of the presence of T2DM. Of note, IR that far precedes the clinical diagnosis of diabetes was demonstrated to be associated with the presence of carotid arteriosclerosis in healthy subjects with normal fasting glucose and normal glucose tolerance [7]. In a large longitudinal study by Rundek et al., IR determined an increased risk of incident stroke in non-diabetic subjects followed for 8.5 years. [16]. Moreover, Åberg D. et al. demonstrated that in non-diabetic patients with ischemic stroke IR was elevated and also related to stroke severity [17].

The IR may increase the risk of CVD also by promoting hypertension, as these conditions have common pathogenetic mechanisms. In particular, they share a dysregulation of sympathetic nervous and renin-angiotensin systems that results in the enhanced stimulation of both adrenergic and angiotensin II receptors, a reduction of nitric oxide with endothelial dysfunction, the enhancement of oxidative stress, and the production of inflammatory cytokines [18,19,20,21]. In a previous work by our group it has been demonstrated that in hypertensive patients the target organ damage, represented by left ventricle hypertrophy and/or carotid atherosclerosis is a predictor of incident diabetes [22]. In light of the tight link between IR and hypertension, we can speculate that in that population, the presence of IR was likely and may have contributed to the incident diabetes.

### 4.3. Insulin Resistance and Coronary Atherosclerosis

As for carotid atherosclerosis, likewise IR increases the risk of coronary atherosclerosis even in the absence of diabetes. In fact, Mossmann et al. in a small cross-sectional study, that included 54 non-obese non T2DM-patients referred to CA for suspected CAD, demonstrated that a high HOMA-IR was associated with significant CAD (stenoses > 50%) [8].

In regard to the relation between the IR and the severity of CAD, this was investigated for the first time in T2DM patients in the study by Srinivasan MP et al. that demonstrated in 61 consecutive patients who underwent CA a significant correlation between log HOMA-IR and severity of coronary atherosclerosis, measured with the Gensini score [23], which takes into account three parameters for each coronary lesion: The severity of obstruction, the location of the lesion in the coronary tree, and the presence of collaterals. 

In the current study, we used the SS I, another widely used score for CAD, that takes into account the complexity of coronary atherosclerotic lesions based on 11 angiographic variables: Dominance, number of diseased segments, total occlusion, trifurcation, bifurcation type and angulation, aorto-ostial lesion, tortuosity, lesion length, heavy calcification, thrombus, and diffuse disease [10]. Hereby, we demonstrated for the first time that in non-diabetic patients undergoing CA, there is a significant correlation between insulin resistance and CAD, with the HOMA index going hand in hand with the SS.

Interestingly, we also found a stronger correlation between HOMA and SS in women than in men, which can be presumably explained by the loss of the protective effect exerted by sex hormones during menopause as in our sample the women mean age was 71 ± 9. This hypothesis is corroborated by the study by Os I. et al. in which short-term treatment with trans-dermal estradiol reduced IR in non-diabetic women with CAD [24].

Furthermore, it is well established that T2DM patients have higher prevalence of three-vessel disease compared to non-diabetic patients [25], however the relation between IR and the number of diseased vessels at CA in non-diabetic patients has never been investigated before. Notably we found that, even when correcting for the presence of smoking, hypertension, or dyslipidemia, HOMA index was the strongest predictor of three-vessel disease. Our results thus corroborate the previous findings that IR contributes to the coronary atherosclerotic process independently of the presence of other comorbidities and independently of the presence of diabetes. Furthermore, in a previous work, we have already demonstrated that in non-diabetic patients referred to CA and with higher SS, the risk of developing T2DM was higher. Therefore, we can speculate that patients in that study with more severe CAD probably had severe IR [26].

### 4.4. Clinical Implications

Diabetes represents a “CAD risk equivalent” [27], therefore the majority of cardiologists when visiting patients with known or new onset diabetes adopt a more careful clinical behavior by programming closer outpatient visits or by giving more aggressive therapy. Insulin resistance is a main component of T2DM and contributes to its detrimental effects, however recent studies demonstrated that IR negatively influences carotid and coronary atherosclerosis even in the absence of diabetes [7,8].

We hereby demonstrated that the higher the IR is, the higher is the likelihood of finding complex and three-vessel disease at CA even in non-diabetic patients. This implies that so far, we have probably underestimated the relevance of IR itself in the risk stratification for CAD and that the HOMA index calculation should be considered for routine use in our clinical practice for the management of non-diabetic patients.

IR is not an irreversible condition and may be counteracted. Thus, to be able to identify IR with normal glycaemia could allow recognition of high-risk patients who are potential candidates to benefit from specific interventions. Indeed, the work by de lima Sanches et al. demonstrated that an interdisciplinary weight-loss program promoted a reduction of the common carotid artery IMT in obese adolescents and the improvement of HOMA-IR was an independent predictor of carotid IMT changes in this population [28]. These results are encouraging and suggest that HOMA-IR in non-diabetic patients not only has the potential to become a standard parameter to stratify the cardiovascular risk, but it also may guide the choice of specific treatments.

### 4.5. Limitations

The current study has several limitations. (1) This was a cross-sectional study. (2) The study sample was relatively small, however because the significance was high it is unlikely that with a larger sample the results would have been different. (3) Follow-up data were not collected; therefore, it is unknown whether in patients with high SS and high HOMA-IR the incidence of diabetes or cardiovascular events is higher. (4) Blood samples for the measurement of insulin and glucose were obtained the day before discharge, therefore we cannot exclude that somehow the acute event in ACS patients may have influenced their levels in the serum.

## 5. Conclusions

Insulin resistance in non-diabetic patients goes hand in hand with the extension and severity of coronary atherosclerosis. The HOMA index is an independent predictor of three-vessel disease at CA. Our findings suggest that the HOMA-IR could be useful for risk stratification of CAD in non-diabetic patients.

## Figures and Tables

**Table 1:** Baseline characteristics.

**Categorical Variables**	**SS1 (*N* 31)**	**SS2 (*N* 29)**	**SS3 (*N* 35)**	**SS4 (*N* 31)**	***p* Value (χ^2^ Test)**
Males, *n*(%)	21 (68)	18 (62)	31 (89)	24 (77)	0.18
Smokers, *n*(%)	8 (26)	12 (41)	17 (49)	8 (26)	0.11
Hypertension, *n*(%)	24 (77)	15 (52)	25 (71)	24 (77)	0.22
Dyslipidemia, *n*(%)	19 (61)	20 (69)	24 (69)	26 (84)	0.24
SCAD, *n*(%)	20 (65)	11 (38)	21 (60)	23 (74)	0.07
SCA, *n*(%)	17 (55)	20 (69)	18 (51)	12 (39)	0.06
**Continuous Variables**	**SS1(*N* 31)**	**SS2 (*N* 29)**	**SS3 (*N* 35)**	**SS4 (*N* 31)**	***p* Value (ANOVA Test)**
Age, yrs	67 ± 9	65 ± 13	64 ± 11	71 ± 11	0.06
BMI, kg/m^2^	27 ± 6	26.8 ± 4	28.5 ± 4	26.8 ± 3	0.31
FPG, mg/dL	99 ± 22	97 ± 13	105 ± 24	101 ± 16	0.33
SBP, mmHg	131 ± 16	131 ± 19	127 ± 16	134 ± 16	0.44
DBP, mmHg	72 ± 8	74 ± 10	72 ± 10	75 ± 11	0.46
EF, %	54 ± 10	51 ± 9	53 ± 9	54 ± 8	0.49
eGFR _EPI-CKD_ mL/min	79 ± 22	81 ± 24	80 ± 21	71 ± 21	0.33

Continuous variables are reported as mean ± SD; Categorical variables are expressed as numbers (percentages). SS1: lowest quartile of syntax score; SS2: second quartiles of syntax score; SS3: third quartile of syntax score; SS4: highest quartile of syntax scoreBMI: Body mass index; FPG: Fasting plasma glucose; SBP: Systolic blood pressure; DBP: Diastolic blood pressure; EF: Ejection fraction; SCAD: Stable coronary artery disease; SCA: Acute coronary syndrome; eGFR: Estimated glomerular filtration rate by the EPI-CKD formula.

**Table 2:** Adjusted binary logistic regression analysis for highest quartile of SS (SS4) and for three-vessel disease.

	SS4				Three-Vessel Disease			
Variables	OR	95% CI		*p*	OR	95% CI		*p*
Males	0.46	0.08	2.49	0.37	0.43	0.09	2.06	0.29
Age	1.12	1.03	1.22	0.01	1.05	0.98	1.13	0.15
HOMA-IR	1.22	1.11	1.34	< 0.01	1.22	1.09	1.36	< 0.01
Hypertension	0.15	0.01	1.67	0.12	0.37	0.05	2.79	0.33
Smoke	2.06	0.35	12.19	0.43	0.41	0.08	2.06	0.28
Dyslipidemia	1.09	0.14	8.19	0.93	0.89	0.14	5.67	0.89
BMI	0.78	0.59	1.02	0.07	0.83	0.66	1.05	0.12
Ca^2+^Antagonists	1.77	0.28	11.05	0.54	1.71	0.31	9.46	0.54
BB	1.27	0.19	8.21	0.8	1.88	0.29	11.90	0.50
Statins	0.21	0.01	5.07	0.34	0.4	0.03	5.52	0.49
ACEI-ARBs	3.36	0.35	31.9	0.29	3.86	0.50	29.7	0.19
LDL	1.01	0.99	1.02	0.41	1	0.98	1.02	0.74

Adjustment for confounding variables was obtained by including simultaneously all the variables in the regression analysis. SS4 indicates the highest quartile of syntax score. SS: Syntax score; BMI: Body mass index; HOMA-IR: Homeostasis model assessment: Insulin resistance; BB: Beta-blockers; ACE-ARBs: Angiotensin converting enzyme inhibitors-angiotensin II receptors blockers; LDL: Low-Density Lipoprotein.
